# Assessing assemblage-wide mammal responses to different types of habitat modification in Amazonian forests

**DOI:** 10.1038/s41598-022-05450-1

**Published:** 2022-02-02

**Authors:** Paula C. R. Almeida-Maués, Anderson S. Bueno, Ana Filipa Palmeirim, Carlos A. Peres, Ana Cristina Mendes-Oliveira

**Affiliations:** 1 Instituto de Ciências Biológicas - LABEV, Universidade Federal do Pará, Belém, PA Brazil; 2 Faculdade Estácio de Castanhal, Castanhal, PA Brazil; 3 Unama Parque Shopping, Belém, PA Brazil; 4 Instituto Federal de Educação, Ciência e Tecnologia Farroupilha, Júlio de Castilhos, RS Brazil; 5 School of Environmental Sciences, University of East Anglia, Norwich, Norfolk UK; 6 CIBIO-InBIO, Universidade do Porto, Campus de Vairão, Rua Padre Armando Quintas, 4485-661 Vairão, Portugal

**Keywords:** Biodiversity, Community ecology, Conservation biology

## Abstract

Tropical forests are being heavily modified by varying intensities of land use ranging from structural degradation to complete conversion. While ecological responses of vertebrate assemblages to habitat modification are variable, such understanding is critical to appropriate conservation planning of anthropogenic landscapes. We assessed the responses of medium/large-bodied mammal assemblages to the ecological impacts of reduced impact logging, secondary regrowth, and eucalyptus and oil palm plantations in Eastern Brazilian Amazonia. We used within-landscape paired baseline-treatment comparisons to examine the impact of different types of habitat modification in relation to adjacent primary forest. We examined assemblage-wide metrics including the total number of species, number of primary forest species retained in modified habitats, abundance, species composition, and community integrity. We ranked all types of habitat modification along a gradient of assemblage-wide impact intensity, with oil palm and eucalyptus plantations exerting the greatest impact, followed by secondary regrowth, and selectively logging. Selectively-logged and secondary forests did not experience discernible biodiversity loss, except for the total number of primary forest species retained. Secondary forests further experienced pronounced species turnover, with loss of community integrity. Considering the biodiversity retention capacity of anthropogenic habitats, this study reinforces the landscape-scale importance of setting aside large preserved areas.

## Introduction

Habitat loss and degradation are currently the primary drivers of biodiversity loss and species turnover worldwide^1,2^. This is particularly pertinent in tropical forests which harbour the highest levels of biodiversity but have succumbed to the steepest deforestation rates^3^, which is particularly illustrated by Amazonian forests^4^. For instance, ~ 25% of the original forest cover throughout the Brazilian Amazon has been converted into a variety of non-native anthropogenic habitats^5^. To make matters worse, biodiversity loss has been further aggravated by marginally detectable patterns of human-induced degradation of the remaining native forests^6,7^. Understanding species responses to varying land-use intensities resulting in different types of forest habitat modification can ensure effective management^8^.

Species diversity in human-modified landscapes is expected to be primarily affected by habitat quality^9^, which is generally higher at sites characterized by low structural and compositional contrasts with native undisturbed ecosystems^6^. Indeed, while degraded forests retaining part of their original structure can still provide habitat for many forest species (e.g., selectively logged, and secondary forests), newly converted anthropogenic habitats (e.g., annual croplands, fast-growing tree plantations and pastures) often cannot provide similar structural and trophic resources and typically host different microclimatic conditions compared to the previous forest habitat^10^. Therefore, changes in native biodiversity are expected to be less drastic in partially degraded rather than in converted habitats^11^, with the former type of habitat disturbance retaining the highest biodiversity value^1,6^. Moreover, local species extinctions are expected to be particularly pronounced in species that cannot persist under novel disturbed habitat conditions^12^. Those species usually correspond to strict forest specialists, and their local extirpation may be offset by the colonization of generalist species that can thrive in open and anthropogenic habitats^13^, further augmenting species compositional differences in newly modified habitats^14^.

Mammal assemblages inhabiting Amazonian forests are typically species-rich, and range in body mass from < 15 g to > 150 kg^15^. Sympatric mammal species often occupy all vertical forest strata, and play several critical functions in ecosystem functioning, including seed dispersal, herbivory, while controling both higher and lower trophic levels^16,17^. Several studies have assessed the effects of habitat modification on mammal assemblages, whose responses are highly variable^1,18^, further diverging among continents^19^. In Amazonian forests, however, most studies are deployed at the local scale and focus on a single type of disturbance (but see^6,20^), hampering direct comparisons between different forms of human disturbance of varying intensity. Nevertheless, such understanding is more critical than ever due to the unprecedented rates of forest conversion into human-modified land^5^, followed by widespread degradation of remaining native forest across the Amazon biome^21^. In particular, medium and large-bodied arboreal species, which often occur at lower densities^22^, are expected to be mostly impacted^23^.

Here, we assess assemblage-wide mammal responses to different forms of anthropogenic disturbance in Amazonian forests. To do so, we synthetised information on medium and large-bodied mammal assemblages across four working landscapes characterized by different land-use types in the Eastern Brazilian Amazon, including two landscapes subjected to forest degradation: selectively logged forest^24^ and secondary forest^25^, and two landscapes subjected to partial forest conversion: oil palm monoculture^26^ and eucalyptus plantations^27^. We first assessed within-landscape mammal responses by comparing mammal assemblages to each modified habitat type with those in an adjacent primary forest baseline. We expected patterns of mammal diversity, overall abundance and species composition to change in all modified habitat types^1,18^. To examine differences in mammal responses to each human-modified habitat type, we then compared mammal responses across landscapes. We hypothesised that mammal assemblages were less diverse in severely modified habitats with the highest structural and compositional contrast^9^. We expected degraded habitats to sustain elevated mammal diversity, particularly forests subjected to reduced-impact selective logging which most resembles an undisturbed primary forest^28^, followed by secondary forests, which amount to native stands under early successional stages^29^. Conversely, mammal diversity is expected to be lowest following clear-cut conversion into oil palm plantations^30^. Our overarching aim is to provide an overview to what extent each of the modified habitat types assessed here is detrimental to mammal assemblages across lowland Amazonia.

## Methods

### Study area

This study is focused on four human-modified landscapes in the Eastern Amazonian states of Pará and Amapá (Fig. 4). Each landscape consisted of a heterogeneous mosaic of primary forest habitat, used as a baseline control, adjacent to one of four different human-modified habitat types: reduced-impact selectively logged forest, second-growth forest, and fast-growing tree monoculture including eucalyptus and oil palm plantations. In all landscapes surveyed, baseline control areas consisted of primary unflooded closed-canopy Amazonian Forest (PF), often referred to as *terra firme* forest*,* characterized by minimal anthropogenic disturbance, canopy height ranging from 20 to 50 m^24–27^ and forest patch sizes ranging between 64,000 (oil palm landscape) and 209,000 ha (logged forest landscape). The eucalyptus plantation landscape was further influenced by a large patch of natural scrubland savannah (*Cerrado*) and PF areas therein are characterized by relatively intact riparian forest habitats^27^.

**Figure 1:**
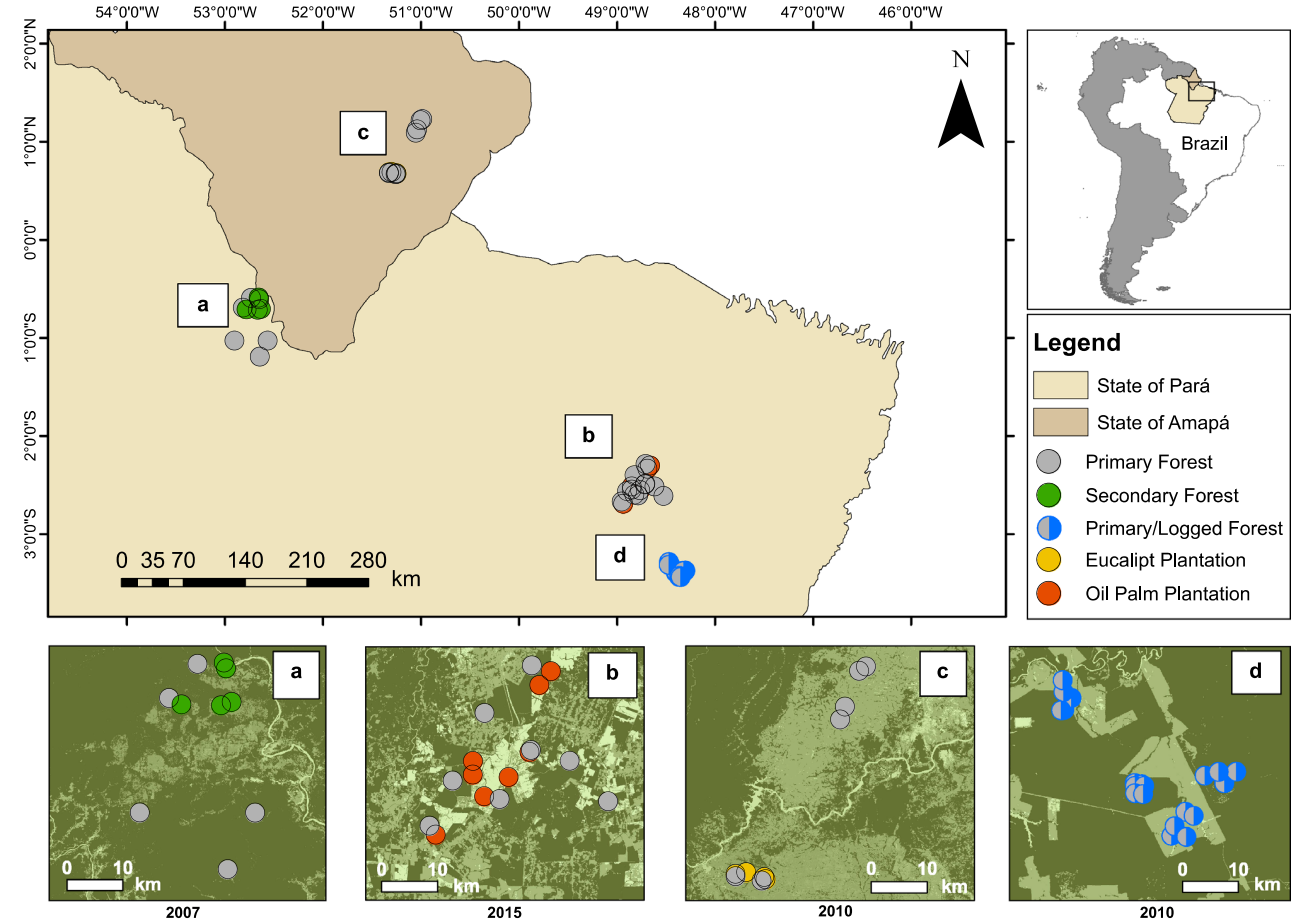
Geographic location of each of the four landscapes where medium to large-bodied mammal assemblages were surveyed in Eastern Brazilian Amazonia. Within each landscape, sampling sites are represented by dots and colour-coded according to habitat type: (**a**) secondary forest (green), (**c**) eucalyptus plantation (yellow), (**d**) oil palm plantation (orange) and adjacent primary forest (grey). As an exception, sampling points in the logged forest landscape (**b**) are half grey-half blue coloured to represent sampling both before and after logging, respectively. Each landscape is amplified to improve clarity in the positional context of sampling sites. In the satellite images (Accessible in https://mapbiomas.org), dark green represents forest areas, with the exception of landscape c, where the Amazon Savannah is predominantly in light green.

The logged forest-landscape is located in Paragominas, Pará (centroid coordinates: 03° 39′ 52″ S, 48° 33′ 46″ W, Fig. 1b) and managed by the timber enterprise *Cikel Brasil Verde* (Keilla Group). This company uses reduced-impact logging (RIL) techniques which aims to minimize collateral damage to the forest structure^31^, including careful opening of roads and selection of target trees^32^. This landscape is divided into several compartments of 2000 to 5000 ha, referred to as Annual Production Units. Complying with RIL legislation, an average of eight large trees per hectare are logged each year in one of these compartments. Each compartment is logged at intervals of 35 years^33^. In this landscape, sampling sites were surveyed twice: one year before the first tree harvest and one year thereafter. To improve text flow, hereafter we refer to the forest prior to first logging as PF and the forest after logging as *logged forest* (LF).

The second-growth forest landscape is located in Almeirim, Pará (01° 30′ 00″ S, 53° 20′ 00″ W, Fig. 1a), where areas of *secondary forest* (SF) occupy 50,000 ha and correspond to native forest that had been regenerating for 14–19 years following abandonment from fast-growing *Gmelina arborea* tree plantations. Sampling sites were characterized by a low-statured (10–15 m) forest structure comprised of arborescent palms, pioneer trees in an advanced stage of succession and dense understorey^25^.

The eucalyptus plantation landscape is located in Porto Grande/Tartarugalzinho, Amapá (00° 42′ 46″ N, 51° 24′ 46″ W, Fig. 1c). In this landscape, native Amazon Savannah were replaced in the 1970s by *Eucalyptus plantations* (EP) (*Eucalyptus urophilla* and *E. tereticornis*). The standard distance between trees is 3 × 2 m, which are clear-cut every 6–7 years. Sampling sites were located in EP that were 3 to 6 years old and characterized by a discontinuous canopy of 10 to 14 m in height^27,34^.

The oil palm plantation landscape is sited at Moju, Pará (00° 42′ 46″ N, 51° 24′ 46″ W, Fig. 1d), within the 103,000-ha *Agropalma* private landholding. In part of this landscape, oil palm monoculture had been planted since the 1980s in areas previously used for cattle pastures and conventional logging^26^. Sampling sites in *oil palm plantation* (OP) (species *Elaeis guineensis*) were characterized by 10–20 years-old palms planted within at a standard 10 × 10 m grid. Similarly to EP, OP canopy hosts a discontinuous canopy cover, and litter distribution was aggregated, so that bare soil was often exposed. In this habitat type, the understorey was open and simplified, with the presence of high-climbing woody lianas^35^.

### Mammal surveys

Medium to large-bodied mammals were surveyed using line transect censuses (LTC), in which two observers walked slowly (~ 1 km/h) along an established transect of variable length. Surveys were carried out early in the morning (05:30 h to 10:30 h) and in the afternoon (16:00 h to 21:00 h) to match the typically bimodal mammal activity rhythm. Transects were alternately surveyed, maintaining a minimum of 24 h sampling interval on the same transect^36^. Since detectability of indirect records such as tracks and scats could differ between the different habitat types covered (e.g., potential higher detectability in tree plantations than in LF or SF), we only considered direct mammal records, including both visual and acoustic cues. All field surveys were carried out between 2002 and 2010 during both the wet and dry seasons.

Each sampling site corresponded to one linear transect. Sampling effort varied between the landscapes surveyed in terms of number, size (transect length), and number of times each sampling site was surveyed. Subsequently, the total distance (km) surveyed also varied between landscapes (Table S1). In the logged forest-landscape, a total of 21 sampling sites were surveyed both before (498 km of census walks) and after (560 km) logging events. A total of 10 sampling sites were surveyed in the secondary forest landscape, five of which located in the adjacent PF (197.7 km) and another five sites in the SF (238.4 km). The eucalyptus plantation landscape was sampled at 10 sites, six of which located in PF (145.2 km) and four in the EP (79.2 km). The oil palm plantation-landscape was sampled at 16 sites, half of which located in PF (310.8 km) and the other half in OP (394.8 km). Additional information on each survey site, including geographic coordinates and sampling effort, can be found in Table S1 (Supplementary Material).

### Data analysis

Data analyses were first carried out within each of the four landscapes surveyed by comparing each human-modified habitat with the adjacent PF baseline. Within-landscape results were then compared across landscapes.

### Within-landscape responses

For each of the eight habitat types (i.e., four modified habitats and four adjacent PF), we examined the *rarefied total species richness* based on sample coverage [as proposed by^37^]. Accordingly, individuals are set as samples and the curves are calculated using the sample coverage estimator, which estimates the proportion of the total number of individuals in an assemblage that belongs to the species represented in the sample. This approach accounts for the fact that species-rich sites require a greater number of individuals to be fully characterized than species-poor sites. Using the same procedure, we also estimated the *rarefied number of primary forest species*, which represents the primary forest species retained within any adjacent anthropogenic habitats. We assumed that all species in the region are originally from primary forests, as the altered habitats were all originally native environments. The significance of observed differences in species richness between habitats was evaluated by visually comparing rarefaction curves and their associated 95% confidence intervals. If the total observed richness of a species-poor habitat fell outside the 95% confidence interval of a more species-rich habitat, then we inferred that the former sample contained significantly fewer species than the latter^6^.

Comparisons of mammal *species abundance* between any modified and adjacent primary forest habitat were performed using a standard t-test, except for the logged forest landscape for which we applied a paired t-test. To do so, we used the standardized species abundance given by the number of records detected per 10 km surveyed for each habitat type per landscape.

*Species composition* was examined using Non-Metric Multidimensional Scaling (NMDS) ordination based on the Bray–Curtis dissimilarity measure using species relative abundance. Within each landscape, differences in *species composition* between human-modified and adjacent PF habitats were further analysed using a Multivariate Permutational Variance Analysis (PERMANOVA) with 1000 permutations as implemented in the ‘adonis’ function in the ‘vegan’ package^38^. The *r*^*2*^ value indicates the effect of each land use change on the species composition, in this case, the higher the *r*^*2*^ value, the greater the change in species composition between PF and altered habitats.

We further calculated mammal *community integrity* which, within each surveyed landscape, considers the degree of similarity (1—dissimilarity) between any human-modified habitat and its adjacent primary forest baseline. *Community integrity* was based on the Bray–Curtis distance measure using species relative abundance and was quantified as the difference between the value of each site and the average of primary forest sites^13^. Accordingly, high values of community integrity indicate that modified habitats resemble primary forest mammal assemblages. Comparisons of the mammal *community integrity* between any modified-habitat and its adjacent PF were performed using a standard t-test, except for the logged forest-landscape for which we applied a paired t-test.

### Responses across landscapes

We compared each of the mammal assemblage-metrics—*rarefied total species richness*, *rarefied number of primary forest species*, *species abundance* and *community integrity* –across the four human-modified habitats. We did so by calculating the mean and 95% confidence intervals of the percentage change in relation to their respective adjacent primary forest baseline.

### Ethical statement

The manuscript contains original data and is not under consideration in another journal.

## Results

A total of 46 mammal species representing 18 families and seven orders were recorded across the four landscapes (*N* = 5245 records; Table S2). In total, 12 species (26%) were only present in *Primary Forest* areas (PF_LF_ = 4 species, PF_SF_ = 5, PF_EP_ = 8, PF_OP_ = 18), whereas three species—*Dasypus kappleri*, *Galictis vittata* and *Saimiri sciureus—*were only present in human-modified habitats (LF = 5 species, SF = 4, EP = 1, OP = 2). Primates comprised the most recorded species, particularly *Sapajus apella* (*N* = 1139 records, 21.7%), *Saguinus ursulus* (*N* = 999, 19.0%) and *Alouatta belzebul* (*N* = 870, 16.6%). The most ubiquitous species were *Mazama americana* (*N* = 180 records, 3.4%), *Tapirus terrestris* (62, 1.2%) and *Eira barbara* (17, 0.3%) which were recorded in 7 of the 8 habitat types surveyed. Seven species were recorded only either once or twice considering all landscapes surveyed.

### Within-landscape responses

**Figure 2:**
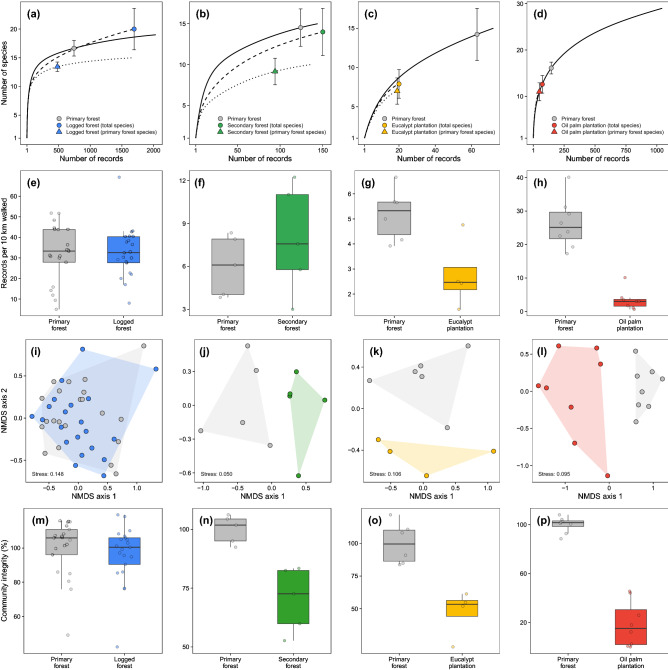
Mammal assemblage-wide metrics for human-modified and adjacent primary forest within each landscape: (**a**–**d**) *rarefied total species richness* in modified habitat and the adjacent primary forest, and *rarefied number of primary forest species* retained in modified habitat; (**e**–**h**) *species abundance* (number of records per 10 km walked); (**i**–**m**) *species composition*, represented in an ordination diagram obtained using the first two NMDS axes; and (**n**–**q**) *community integrity*, defined as the degree of similarity between disturbed and undisturbed habitats. Within each landscape, sampling sites are represented by dots and colour-coded according to habitat type: logged forest (blue), secondary forest (green), eucalyptus plantation (yellow), oil palm plantation (red) and adjacent primary forest (grey). As an exception, dots/triangles in (**a**–**d**) represent the rarefied number of species within each habitat type per landscape, which was obtained by further considering the number of records and corresponding sample coverage (see details in the Data Analysis).

The *rarefied total species richness* was similar between either *logged forest* (LF) (estimate [95% CI] 20.0 [16.4–23.6]) or *secondary forest* (SF) (14.0 [11.1–16.9]) and their adjacent PF sites (16.7 [15.3 –18.0] and 14.5 [12.2–16.8], respectively). Conversely, the *rarefied number of primary forest species* in both LF (13.4 [12.6–14.3]) and SF sites (9.2 [7.5–10.8]) was lower than in their adjacent PF sites (Fig. 1a,b). The *rarefied total species richness* was drastically reduced in landscapes that succumbed to complete forest replacement, with *eucalyptus plantation* (EP) (7.9 [6.1–9.7]) and *oil palm plantation* (OP) (12.6 [10.7–14.5]) sustaining fewer species than their adjacent PF sites (14.2 [10.9–17.5] and 16.1 [14.8–17.4], respectively; Fig. 2c,d). This was also observed in terms of the *rarefied number of primary forest species* following forest conversion into tree plantations (EP = 7.0 [5.3–8.7], OP = 11.0 [9.1–12.9]; Fig. 1c,d).

Likewise, *species abundance* was similar between either LF (mean ± SD: *N* = 32.8 ± 12.5 records/10 km) or SF (*N* = 7.9 ± 3.8) and their adjacent PF sites (LF: *N*_*PF*_ = 32.0 ± 14.0, *t* = –0.259, *P* = 0.798; SF: *N*_*PF*_ = 6.0 ± 2.1, *t* = –0.969, *P* = 0.368; Fig. 1e,f). Following full forest conversion, species abundance was lower in both EP (*N* = 2.8 ± 1.4) and OP (*N* = 3.4 ± 3.0) compared to their adjacent PF sites (EP: *N*_*PF*_ = 5.2 ± 1.0, *t* = 2.918, *P* = 0.032; OP: *N*_*PF*_ = 26.2 ± 7.3, *t* = 8.183, *P* < 0.001; Fig. 1g,h).

Multivariate patterns of *species composition* following forest degradation by logging overlapped that in adjacent PF (PERMANOVA: *r*^2^ = 0.022, *P* = 0.496; Fig. 1i), whereas species composition was highly divergent in SF (*r*^2^ = 0.356, *P* = 0.012), EP (*r*^2^ = 0.264, *P* = 0.012) and OP (*r*^2^ = 0.443, *P* = 0.001) compared to their respective adjacent PF sites (Fig. 1j,l). These results were also reflected in the degree to which mammal assemblages were dissimilar between neighbouring disturbed and undisturbed sites (Fig. 1m,p). Indeed, *community integrity* was similar in logged and unlogged forest (LF: mean ± SD: 96.5 ± 17.0; PF_LF_ = 100.0 ± 16.6, *t* = 0.671, *P* = 0.506; Fig. 1m), but it was considerably lower in SF (PF_SF_ = 100.0 ± 6.0, SF = 70.2 ± 13.7,; *t* = 4.464, *P* = 0.005; Fig. 1n), EP (PF_EP_ = 100.0 ± 15.7, EP = 47.2 ± 18.0; *t* = 4.769, *P* = 0.003; Fig. 1o) and OP (PF_OP_ = 100.0 ± 6.4, OP = 18.6 ± 18.5; *t* = 11.745, *P* < 0.001; Fig. 1p) compared to their adjacent primary forest baselines.

### Responses across landscapes

Differences among the four anthropogenic habitat types were examined for each assemblage-wide metrics as the percentage change in relation to their respective primary forest baseline (Fig. 2). In terms of *rarefied species richness*, LF was more species-rich compared to the corresponding PF baseline (Fig. 2a). All remaining modified habitats harboured fewer species than their corresponding baselines, but this was significantly lower only for tree plantations (OP and EP; Fig. 2a). Considering the *rarefied number of primary forest species*, all modified habitats harboured fewer species than their adjacent baselines, but LF presented the smallest differences compared to SF, EP and OP (Fig. 2b). *Overall abundance* was only significantly lower within OP (Fig. 2c). Overall *community integrity* in logged and unlogged forest was similar, whereas the difference of this metric was increasingly higher for second-growth and tree plantations compared to their adjacent baselines (Fig. 2d).

**Figure 3:**
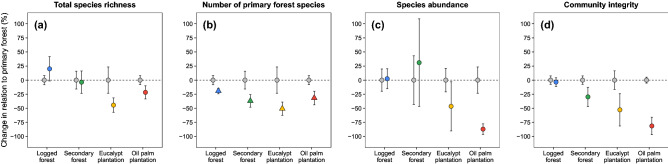
Percentage changes (mean ± 95% CIs) in assemblage-wide metrics within anthropogenic habitats compared to their adjacent primary forest baselines: (**a**) *rarefied species richness*, (**b**) *rarefied number of primary forest species*, (**c**) *overall abundance,* and (**d**) *community integrity*. Triangles indicate metrics when only primary forest species were included and are colour-coded according to habitat type: primary forest (grey), logged forest (blue), secondary forest (green), eucalyptus plantation (yellow) and oil palm plantation (red).

Compositional differences in assemblages can be further illustrated by the degree to which mammal species occur along the 78 transects sampling multiple habitat types (Fig. 3). Species ordered at the bottom were often relatively abundant but largely restricted to undisturbed primary forest, whereas the overall species incidence was gradually thinned out in increasingly disturbed habitats. Of the 13 species of primates recorded, four were exclusive to primary forests, and the same was observed with other strictly arboreal species, such as *C. didactylus* and *P. flavus* (Fig. 3; Figure S1). In addition, some essentially carnivorous species, as *L. pardalis*, *L. wiedii*, *P. concolor*, and *P. yagouaroundi*, were also exclusive to the PF (Fig. 3; Figure S1).

While primary forest contained most species absent from any of the anthropogenic habitats, logged and secondary forest often harboured a fraction of forest specialists (e.g. *Priodontes maximus* in LF and *Saimiri sciureus* in SF). Finally, both eucalyptus and oil palm plantations retained highly depauperate mammal assemblages, and at that primarily habitat generalists or wide-ranging species that likely also used adjacent primary forest (e.g. *Cerdocyon thous* and *Dasypus novemcinctus*) (Fig. 3; Figure S1).

## Discussion

Rapid anthropogenic modification of tropical forest habitats has led to unprecedented rates of population loss in terrestrial vertebrates^18,39^. Under this context, we integrate data from four independently surveyed landscapes using a standardized approach to document consistently negative mammal responses across a broad spectrum of structural change in novel anthropogenic habitats. The effects of complete forest habitat conversion were clearly more severe than those of habitat degradation. In fact, the number of species in either logged forest (LF) or secondary forest (SF) was only lower than that in adjacent primary forest (PF) when the number of primary forest species retained in degraded habitats was considered. Considering different forms of habitat degradation, mammal responses to logging were less severe than those to second growth (i.e., higher number of species and community integrity in LF). Considering commercial tree plantations, eucalyptus (EP) and oil palm (OP) monoculture performed similarly in terms of species retention. In relation to their respective baselines, EP harboured higher levels of overall abundance and community integrity, while OP retained higher species richness in terms of both the entire assemblage and primary forest species.

Undisturbed tropical forests typically exhibit complex vertical stratification, including large emergent trees^40^. Human-induced disturbances often severely simplify forest structure, even in the case of low-intensity disturbance such as reduced-impact logging^1,41^. This explains the consistently lower overall mammal species diversity observed, at least of forest specialists^2,13^. Notwithstanding, mammal responses were less severe within degraded habitat types (LF and SF) than in those converted into tree monoculture (EP and OP). Although habitat degradation drives a simplification in forest structure (e.g., low understorey tree density, absence of woody lianas, thinning of large canopy trees and heavy epiphytic loads^42^, such changes are not as drastic as those induced by complete stand replacement with cropland and pasture. This drastically reduces the spectrum and availability of trophic and structural resources of old-growth forests (e.g., food and shelter^43^, and induces elevated temperatures and lower humidity^10^. Our results echo previous findings showing increasingly detrimental responses to gradually more intensive patterns of land-use change^9^, which has been shown for primates^44^, herpetofauna^45^ and terrestrial biodiversity in general^18^.

**Figure 4:**
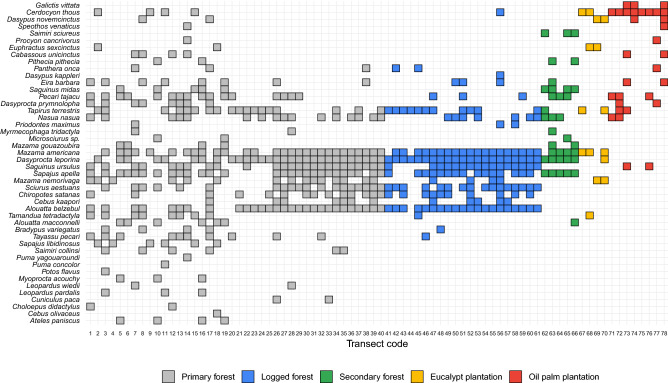
Species recorded at each sampling site across all four human-modified forest landscapes. At each sampling site (transect), species incidence is denoted by a square that is colour-coded according to habitat type (see legend). Line-transects are ordered left to right within habitat type from most to least species rich.

Our results, however, partly contradict a global meta-analysis of the biotic effects of tropical forest disturbance which reported relatively mild changes in mammal assemblages between selectively logged and unlogged forests^1^. Although selectively logging was the least detrimental form of habitat modification, our before-and-after study shows that over 20% of all mammal species found in adjacent PF sites had been apparently extirpated within 1 year after logging. However, the lack of a Before-After Control-Impact (BACI) sampling design^46^ makes our inferences more conservative at this site. In addition to the severity of habitat modification, mammal persistence in anthropogenic habitats further depends on species morphoecological traits^47^. While some species may even benefit from habitat disturbance, particularly small-bodied habitat-generalists, forest specialists tend to be driven to local extinction^2,13^. This is particularly the case of large-bodied arboreal mammals^16,23^, and matches our observation of multiple primate species declining in abundance particularly in oil palm plantations (see Fig. 4; Table S2). Indeed, strictly arboreal species are expected to be most severely affected by changes in forest structure^17^. Detection rates of arboreal species depend on the habitat structure and census technique^48^. In particular, the single use of terrestrial camera-trapping incurs a bias in detection rates against arboreal species, underestimating the negative responses to tropical forest disturbance^47^. Studies based on terrestrial camera-trapping alone overlook important changes in the arboreal mammal fauna, partly explaining the weak mammal responses to forest disturbance observed elsewhere [e.g.^49^]. In this study, we considered only data collected on the basis of line-transect censuses on foot, which ensures the highly effective detectability of the most vulnerable arboreal fauna^50^. However, some other species, rare or less detectable by the sighting, such as felids and canids, may have been under-sampled or hidden in the census. In our study, we observed that some felines considered essentially carnivores were also exclusive to primary forests, which can be partly explained by the greater ecological demand of these species^51^, but also by their low detection through the methodology used. Thus, the use of different types of methods to access the mammal fauna could demonstrate more contrasting data between anthropogenic habitats and their paired forests^26^.

Selective logged forests harboured the highest species richness and were the only modified habitat type sustaining a species composition similar to that of its PF baseline. Although treefall gaps generated by low-impact logging operations also alter the structure of the original forest, this is widely considered one of the most benign forms of extractive land-use for tropical forest biodiversity^1,52^. In the Amazon, microclimatic conditions are known to recover relatively fast from selective logging^53^. Short-term effects of selective logging on Amazonian forest mammals have rarely been detected^54–56^, even in hit-and-run illegal logging within protected areas^57^. Nevertheless, while total species richness was higher in logged forest compared to adjacent unlogged forest, the number primary forest species was higher in the latter, suggesting that some forest specialists tend to decline whereas generalists and open-habitat species tend to increase. Moreover, we only considered the short-term effects of selective logging (i.e. 1 year post-logging). Long-term effects may include additional population declines [but see^28^] and eventually species losses as the extinction debt is paid^58^, which is further exacerbated by the compositional decay in tree assemblages over half a century^59^.

Secondary forests experienced the second lowest difference in species richness and overall abundance in relation to adjacent old-growth. However, early successional forests diverged in their species composition and community integrity. Although second-growth habitats are critical to the persistence of forest species in human-modified landscapes^29^, biophysical and compositional properties fail to converge with those of primary forests even after 25 years of regrowth and remain characterized by a hyper-abundance of pioneer trees^60^. This explains the similar overall species richness, but reduced number of primary forest species and community integrity recorded in the 14–19 years-old secondary forests examined here. Differences in species composition with adjacent PF are likely due to a delay in recovery of forest specialists^61^. While current evidence worldwide attributes a relatively high conservation value to tropical secondary forests^29,62^, we emphasize the limited contribution of this habitat type in terms of composition profiles^63^, which were comparatively more detrimental than those of selective logging^1^.

Both fiber (EP) and biofuel (OP) tree monoculture clearly retained the most species-poor mammal assemblages, accounting for between 47 and 55% fewer species than their adjacent baselines, and 71–92% in terms of overall abundance. Compared to old-growth forest, tree plantations amount to non-native homogeneous habitats in which microclimatic conditions are far more hostile. For example, daytime temperatures in oil palm plantations become 6.5 °C hotter than in primary forest^64^. The native plantation undergrowth in our study areas was also frequently cleared by herbicidal treatments to maximize crop yields. Given such drastic differences^65^, mammal responses observed here were in agreement with the prevailing evidence across tropical forest landscapes^18^. Furthermore, while mammal community integrity in oil palm plantations was lower than that in eucalyptus stands, the number of primary forest species retained in former was higher than that in the latter. Therefore, we attribute a slightly higher conservation value to EP over OP due to the higher species similarity of the former compared to primary forest. Eucalyptus plantations generally support low^66^ to moderate levels of biodiversity^67^, and are primarily occupied by habitat generalists^20^ particularly in young plantations^68^. Biotic responses to eucalyptus monoculture were variable for different taxonomic groups but particularly mild for several invertebrate taxa^6^. Our results reinforce the notion that both types of tree plantations considered here amount to highly detrimental impacts on native biodiversity, particularly under a hostile landscape context where large areas of neighbouring primary forest are no longer available. In Southeast Asia, where oil palm is extensively planted in previously forested areas, only a few native vertebrate species were reported to use these plantations^65^. Despite the presence of some epiphytes and their associated species^69^, oil palm plantations are extensively managed from clear-cuts lacking overstorey shade trees, severely limiting the capacity to even vaguely mimic a closed-canopy forest^70^. This is particularly alarming given that oil palm produces the world's most-consumed vegetable oil and has been predicted to vastly expand in lowland Amazonia^71^.

Reconciling economic development with biodiversity conservation in Amazonian forestlands implies prioritizing economic activities that induce the least amount of structural forest habitat change, which then will hopefully lead to the least detrimental effects on species assemblages^9^. In our study, we found that Amazonian mammal communities within any anthropogenic habitat type do not closely resemble those in adjacent areas of largely intact primary forests. We therefore recommend setting aside large blocks of primary forest as the best strategy to maintain the full complement of vertebrate species and integrity of ecosystem functions within any given working landscape^72^. This could be accomplished by creating networks of protected areas that interconnect old-growth forests with private forests within the wider countryside landscape^8^. However, our findings also indicate that reduced impact logging is a preferred option over silviculture of either eucalyptus or oil palm, but this ignores opportunity costs in terms of land-use revenues. In addition, given the relatively high conservation value of secondary forests in terms of the mammal species richness and abundance, this habitat type could be managed as a safety-net against the impacts of old-growth forest loss. Thus, in a context of high anthropogenic pressure, where it is no longer possible to preserve large blocks of Primary Forest, as is the case in most of the extreme northeast of the Amazon Region, Secondary Forests can be an alternative for expanding protected areas, while low-impact logging can be considered an economic activity that still keeps the forest standing, with a certain degree of biodiversity maintenance. We draw attention to the need for medium and long-term studies to better understand the persistence of effects of the reduced impact logging. We understand that eucalypt or oil palm forestry, despite being commercially considered as forest crops, are incapable of replacing native forests in public policies for the conservation of biodiversity, while secondary or reduced impact logging forests could be acceptable as part of the biodiversity conservation strategies in the Amazon, especially in a context of high anthropogenic pressure. An adequate assessment of mammal assemblage responses to tropical forest habitat modification should also consider medium and large-bodied arboreal species, which tend to be highly vulnerable to forest canopy fracture but severely under-sampled by camera-trapping^47^. The efficient adoption of these strategies by local to regional governments would contribute to minimise tropical forest biodiversity loss.

## Supplementary Information

Supplementary Information 1.

Supplementary Information 2.

Supplementary Information 3.

Supplementary Tables and Figure.

## Data Availability

The datasets generated during and/or analysed during the current study are available from previous publications indicated in the main text.
