# Supplementary material for: Assessing assemblage-wide mammal responses to different types of habitat modification in Amazonian forests
Source: Sci Rep. 2022 Feb 2;12:1797. doi: 10.1038/s41598-022-05450-1 (PMC8810785; doi:10.1038/s41598-022-05450-1)
Supplement: Supplementary file 4 — Supplementary Tables and Figure. [file 41598_2022_5450_MOESM4_ESM.pdf]

Supplementary Material

Assessing assemblage-wide mammal responses to different types of habitat modification in Amazonian forests

Paula C. R. Almeida-Maués, Anderson S. Bueno, Ana Filipa Palmeirim, Carlos A. Peres and Ana Cristina Mendes-Oliveira

Table S1. Details on each sampling site used to survey mid-size and large mammal assemblages across four human-modified landscapes in the Pará (PA) and Amapá (AP) states, Eastern Brazilian Amazonia. One of the landscapes surveyed consisted of 21 sampling sites located in a concessionaire forest selectively logged every 35 years. In that landscape, sampling sites were surveyed one year before (PFLF) and one year after the logging (LF). The remaining three landscapes surveyed consisted of one of either of the human-modified habitat types – second-growth forest (SF), eucalypt plantation (EP) and oil palm plantation (OP) – and an adjacent primary forest (PFSF, PFEP and PFOP). Name of the municipality, geographic coordinates (latitude and longitude in decimal degrees) and sampling effort (total number of meters surveyed) are indicated for each transect surveyed. Transects are coded as in Fig. 4.

Transect code	Habitat	Municipality	Latitude	Longitude	Sampling effort (m)
1	PF_{OP}	Moju	–2.670	–48.951	46200
2	PF_{EP}	Tartarugalzinho	1.129	–51.041	24000
3	PF_{OP}	Moju	–2.606	–48.784	50400
4	PF_{EP}	Tartarugalzinho	1.223	–51.004	6000
5	PF_{SF}	Almeirim	–0.591	–52.736	54500
6	PF_{SF}	Almeirim	–1.026	–52.567	47000
7	PF_{OP}	Moju	–2.611	–48.525	50400
8	PF_{OP}	Moju	–2.287	–48.707	42000
9	PF_{EP}	Tartarugalzinho	1.234	–50.986	14400
10	PF_{SF}	Almeirim	–0.691	–52.819	52750
11	PF_{EP}	Tartarugalzinho	1.095	–51.054	40800
12	PF_{OP}	Moju	–2.489	–48.709	50400
13	PF_{OP}	Moju	–2.401	–48.820	46200
14	PF_{OP}	Moju	–2.562	–48.896	54600
15	PF_{EP}	Porto Grande	0.672	–51.260	26400
16	PF_{SF}	Almeirim	–1.191	–52.648	48050
17	PF_{OP}	Moju	–2.515	–48.616	54600

18	PF_{EP}	Porto Grande	0.684	–51.329	33600
19	PF_{SF}	Almeirim	–1.026	–52.905	36100
20	PF_{LF}	Paragominas	–3.383	–48.402	8000
21	PF_{LF}	Paragominas	–3.384	–48.395	12000
22	PF_{LF}	Paragominas	–3.386	–48.402	8000
23	PF_{LF}	Paragominas	–3.386	–48.393	16000
24	PF_{LF}	Paragominas	–3.393	–48.401	12000
25	PF_{LF}	Paragominas	–3.394	–48.394	16000
26	PF_{LF}	Paragominas	–3.312	–48.470	53000
27	PF_{LF}	Paragominas	–3.300	–48.463	38000
28	PF_{LF}	Paragominas	–3.294	–48.471	26000
29	PF_{LF}	Paragominas	–3.283	–48.472	24000
30	PF_{LF}	Paragominas	–3.411	–48.352	28000
31	PF_{LF}	Paragominas	–3.415	–48.344	25000
32	PF_{LF}	Paragominas	–3.312	–48.473	47000
33	PF_{LF}	Paragominas	–3.383	–48.315	19000
34	PF_{LF}	Paragominas	–3.372	–48.303	35000
35	PF_{LF}	Paragominas	–3.372	–48.320	39000
36	PF_{LF}	Paragominas	–3.376	–48.334	39000
37	PF_{LF}	Paragominas	–3.372	–48.320	31000
38	PF_{LF}	Paragominas	–3.434	–48.366	21000
39	PF_{LF}	Paragominas	–3.425	–48.363	30000
40	PF_{LF}	Paragominas	–3.435	–48.351	33000
41	LF	Paragominas	–3.383	–48.402	37500
42	LF	Paragominas	–3.384	–48.395	10000
43	LF	Paragominas	–3.386	–48.402	10000
44	LF	Paragominas	–3.386	–48.393	7500
45	LF	Paragominas	–3.393	–48.401	10000
46	LF	Paragominas	–3.394	–48.394	15000
47	LF	Paragominas	–3.312	–48.470	51000
48	LF	Paragominas	–3.300	–48.463	37000
49	LF	Paragominas	–3.294	–48.471	30000
50	LF	Paragominas	–3.283	–48.472	18000
51	LF	Paragominas	–3.411	–48.352	26000
52	LF	Paragominas	–3.415	–48.344	29000
53	LF	Paragominas	–3.312	–48.473	20000
54	LF	Paragominas	–3.383	–48.315	29000
55	LF	Paragominas	–3.372	–48.303	23000
56	LF	Paragominas	–3.340	–48.324	33000
57	LF	Paragominas	–3.376	–48.334	21000
58	LF	Paragominas	–3.372	–48.320	39000
59	LF	Paragominas	–3.434	–48.366	22000
60	LF	Paragominas	–3.425	–48.363	14000

61	LF	Paragominas	–3.435	–48.351	16000
62	SF	Almeirim	–0.712	–52.667	25100
63	SF	Almeirim	–0.702	–52.636	44150
64	SF	Almeirim	–0.709	–52.783	60050
65	SF	Almeirim	–0.604	–52.653	31200
66	SF	Almeirim	–0.587	–52.659	37200
67	EP	Porto Grande	0.676	–51.250	21600
68	EP	Porto Grande	0.682	–51.253	28800
69	EP	Porto Grande	0.690	–51.329	12000
70	EP	Porto Grande	0.694	–51.301	16800
71	OP	Moju	–2.691	–48.936	46200
72	OP	Moju	–2.554	–48.762	46200
73	OP	Moju	–2.301	–48.661	46200
74	OP	Moju	–2.334	–48.689	46200
75	OP	Moju	–2.516	–48.848	16800
76	OP	Moju	–2.494	–48.713	46200
77	OP	Moju	–2.599	–48.821	16800
78	OP	Moju	–2.548	–48.848	46200

Table S2. Number of records (visual and acoustic sights) per mammal species and its overall abundance (%) recorded in each of the human-modified habitat types surveyed – logged forest (LF), second-growth forest (SF), eucalypt plantation (EP), and oil palm plantation (OP) – and an adjacent primary forest habitat (PFLF, PFSF, PFEP and PFOP).

Order	Species	PF$_{LF}$	LF	PF$_{SF}$	SF	PF$_{EP}$	EP	PF$_{OP}$	OP	%
**Cingulata**										
	Dasypus novemcinctus	0	0	0	0	0	2	5	2	0.17
	Dasypus kappleri	0	1	0	0	0	0	0	0	0.02
	Euphractus sexcinctus	0	0	0	0	4	2	0	0	0.11
	Priodontes maximus	0	2	0	0	0	0	2	0	0.08
	Cabassous unicinctus	0	0	0	0	0	0	4	2	0.11
**Pilosa**										
	Bradypus variegatus	0	1	0	0	0	0	5	0	0.11
	Choloepus didactylus	0	0	0	0	0	0	3	0	0.06
	Myrmecophaga tridactyla	2	0	0	1	0	0	1	0	0.08
	Tamandua tetradactyla	0	1	0	0	1	1	15	0	0.34
**Primates**										

Order	Species	PF_{LF}	LF	PF_{SF}	SF	PF_{EP}	EP	PF_{OP}	OP	%
	Cebus kaapori	42	66	0	0	0	0	1	0	2.08
	Cebus olivaceus	0	0	0	0	1	0	0	0	0.02
	Pithecia pithecia	0	0	2	2	0	0	0	0	0.08
	Sapajus apella	509	319	11	29	0	0	271	0	21.72
	Sapajus libidinosus	0	0	0	0	22	0	0	0	0.42
	Saimiri collinsi	9	0	0	0	0	0	38	0	0.90
	Saimiri sciureus	0	0	0	6	0	0	0	0	0.11
	Saguinus midas	0	0	18	34	2	0	0	0	1.03
	Saguinus ursulus	389	325	0	0	1	0	275	9	19.05
	Chiropotes satanas	102	115	0	0	0	0	23	0	4.58
	Alouatta belzebul	328	391	0	0	0	0	151	0	16.59
	Alouatta macconnelli	0	0	22	1	1	0	0	0	0.46
	Ateles paniscus	0	0	25	0	0	0	0	0	0.48
**Carnivora**										
	Leopardus pardalis	0	0	1	0	1	0	1	0	0.06
	Leopardus wiedii	1	0	0	0	0	0	1	0	0.04
	Puma concolor	0	0	0	0	2	0	0	0	0.04
	Puma yagouaroundi	0	0	0	0	0	0	1	0	0.02
	Panthera onca	1	2	0	0	0	0	2	1	0.11
	Cerdocyon thous	0	1	0	0	11	3	0	34	0.93
	Speothos venaticus	0	0	0	0	0	0	1	1	0.04
	Eira barbara	2	4	2	2	1	0	4	2	0.32
	Galictis vittata	0	0	0	0	0	0	0	3	0.06
	Nasua nasua	133	65	0	6	0	0	59	15	5.30
	Potos flavus	0	0	0	0	0	0	1	0	0.02
	Procyon cancrivorus	0	0	0	0	0	0	1	7	0.15
**Perissodactyla**										
	Tapirus terrestris	20	24	0	1	7	2	6	2	1.18
**Artiodactyla**										
	Pecari tajacu	21	10	11	4	0	0	23	4	1.39
	Tayassu pecari	44	1	1	0	0	0	21	0	1.28
	Mazama americana	61	68	13	19	6	8	5	0	3.43

Order	Species	PF_{LF}	LF	PF_{SF}	SF	PF_{EP}	EP	PF_{OP}	OP	%
	Mazama nemorivaga	15	9	0	0	3	2	3	0	0.61
	Mazama gouazoubira	0	0	11	3	0	0	0	0	0.27
**Rodentia**										
	Sciurus aestuans	55	71	1	0	0	0	13	0	2.67
	Microsciurus sp.	0	0	2	1	0	0	0	0	0.06
	Cuniculus paca	3	0	0	0	0	0	0	0	0.06
	Dasyprocta prymnolopha	0	0	0	0	0	0	107	5	2.14
	Dasyprocta leporina	298	217	19	41	9	1	0	0	11.15
	Myoprocta acouchy	0	0	5	0	0	0	0	0	0.10

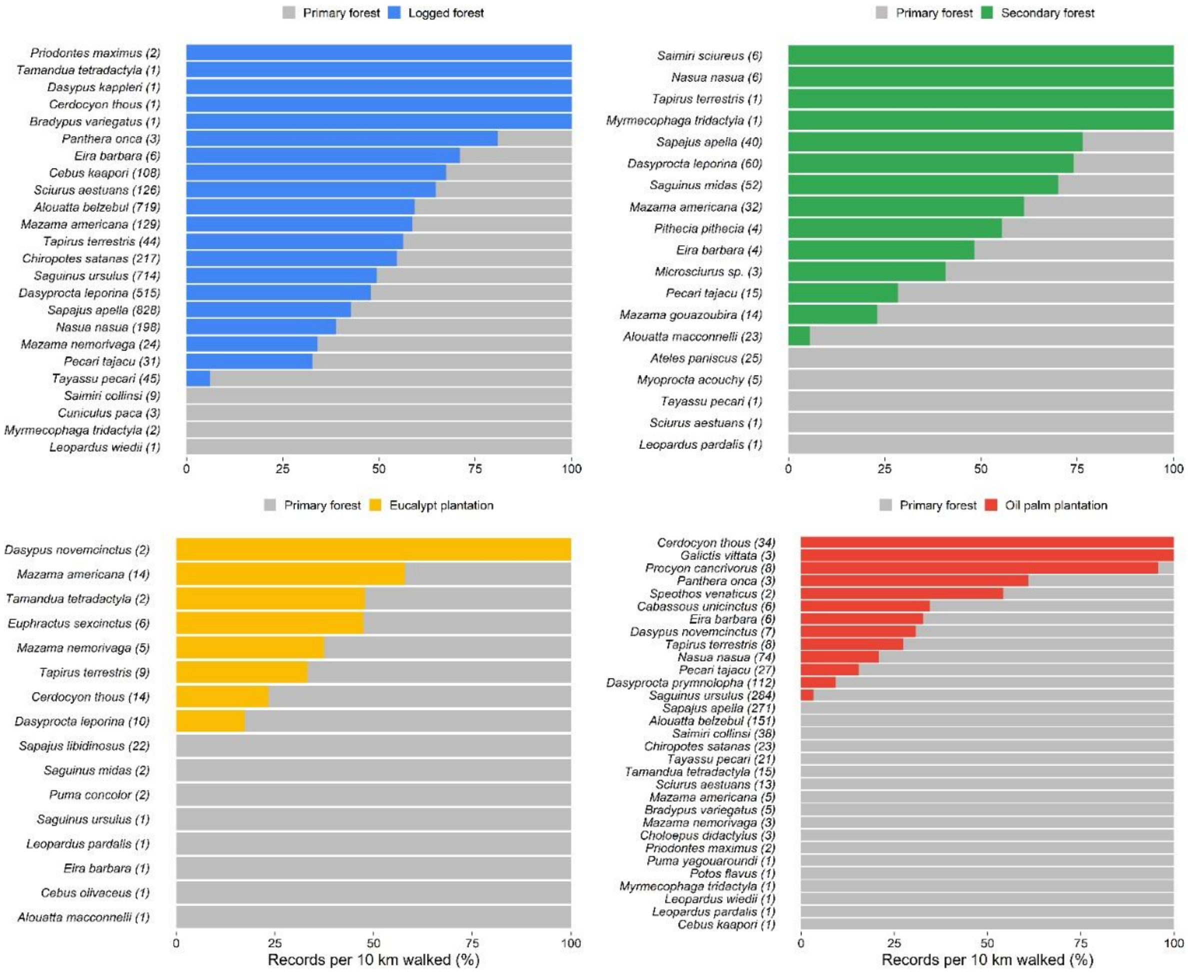

Figure S1. Comparison of the composition and relative abundance of species between each studied anthropogenic habitat and its paired primary forest.
